# Draft Genome Sequences of Chryseobacterium lactis NCTC11390^T^ Isolated from Milk, Chryseobacterium oncorhynchi 701B-08^T^ from Rainbow Trout, and Chryseobacterium viscerum 687B-08^T^ from Diseased Fish

**DOI:** 10.1128/genomeA.00628-18

**Published:** 2018-06-28

**Authors:** Jin-Ju Jeong, Ye Ji Lee, Duleepa Pathiraja, Byeonghyeok Park, In-Geol Choi, Ki Deok Kim

**Affiliations:** a Department of Biosystems and Biotechnology, Laboratory of Plant Disease and Biocontrol, Department of Biosystems and Biotechnology, Korea University, Seoul, South Korea; b Department of Biotechnology, Korea University, Seoul, South Korea

## Abstract

The genus Chryseobacterium, belonging to the family Flavobacteriaceae, contains Gram-negative, yellow-pigmented, rod-shaped, and non-spore-forming bacterial species, which may be free living or parasitic. Here, we report draft genome sequences of type strains of three species of Chryseobacterium containing genes related to biological control and plant growth promotion.

## GENOME ANNOUNCEMENT

The genus Chryseobacterium belongs to the family Flavobacteriaceae and was subdivided from the genus Flavobacterium in 1994 (1). Currently, 109 species of Chryseobacterium have been reported, and some of them exhibit not only plant growth promotion but also biocontrol activities against plant pathogens (2–5). Previously, Chryseobacterium sp. strain ISE14 exhibited plant growth promotion and biocontrol activities against soilborne Phytophthora capsici and airborne Colletotrichum acutatum on pepper plants (5, 6). According to phylogenetic analysis for the identification of strain ISE14, this strain was closely grouped with Chryseobacterium lactis NCTC11390^T^ (7), Chryseobacterium oncorhynchi 701B-08^T^ (8), Chryseobacterium ureilyticum F-Fue-04IIIaaaa^T^ (9), and Chryseobacterium viscerum 687B-08^T^ (10). These species may also contain similar genes related to biocontrol activity, as observed in strain ISE14 (5, 11). Here, we report the draft genome sequences of three type strains of Chryseobacterium species, except for C. ureilyticum F-Fue-04IIIaaaa^T^, whose genome sequence is already deposited in NCBI.

Genomes of strains NCTC11390^T^, 701B-08^T^, and 687B-08^T^ were sequenced using the Illumina MiSeq platform at the Computational and Synthetic Biology Laboratory, Korea University (Seoul, South Korea). Totals of 816,214, 661,893, and 579,742 paired-end reads (87.5-, 82.82-, and 61.09-fold coverage, respectively) for strains NCTC11390^T^, 701B-08^T^, and 687B-08^T^, respectively, were generated, with 500-bp inserts from paired-end sequencing of the genomic library. Low-quality reads were trimmed with a quality threshold of Q20; the trimmed reads were then subjected to *de novo* assembly using the SPAdes assembler (12). The reads were assembled to 36 (NCTC11390^T^), 45 (701B-08^T^), and 60 (687B-08^T^) scaffolds, with the total lengths and G+C contents shown in Table 1. The maximum lengths and *N*_50_ values of the contigs were 1,128,757 and 584,344 bp for NCTC11390^T^, 1,271,342 and 735,093 bp for 701B-08^T^, and 791,600 and 543,073 bp for 687B-08^T^, respectively. Genomes were annotated by the NCBI Prokaryotic Genome Annotation Pipeline (PGAP) service. In total, 4,899, 4,323, and 5,029 coding sequences of strains NCTC11390^T^, 701B-08^T^, and 687B-08^T^ exhibited 84.65, 85.06, and 85.52% sequence similarities with known genes in the NCBI database, respectively. Furthermore, the number of retrieved tRNA, 5S rRNA, 16S rRNA, and 23S rRNA sequences of the strains are shown in Table 1.

**TABLE 1:** Summary of genome sequencing and GenBank accession and version numbers of the type strains of three Chryseobacterium species

Type strain	Genome size (bp)	G+C content (%)	No. of coding sequences	No. of tRNAs	No. of rRNAs by gene type			Accession no.	Version no.
					5S	16S	23S		
C. lactis NCTC11390^T^	5,593,731	36.1	4,899	75	2	1	1	PPEH00000000	PPEH01000000
C. oncorhynchi 701B-08^T^	4,794,425	35.2	4,323	71	1	1	1	PPEI00000000	PPEI02000000
C. viscerum 687B-08^T^	5,693,782	36.2	5,209	73	1	1	1	PPEG00000000	PPEG02000000

As observed in strain ISE14 (11), strains NCTC11390, 701B-08, and 687B-08 contained the genes related to plant growth promotion, such as siderophore, photosynthesis, nitrogen fixation, and phosphate solubilization (13). Biotic and abiotic stress management genes (e.g., catalase, peroxidase, superoxide dismutase, and antibiotic ABC transporter ATP-binding protein) were identified (14–16). In addition, genes involved in gliding motility, which may be a significant factor in the colonization of plant roots, were present. In general, a few differences were observed in the number of genes identified in this study, but many genes were similar in the examined strains, including ISE14. In conclusion, the genomes of three Chryseobacterium spp. will be helpful to our understanding of the plant growth promotion and biocontrol activities of these strains.

### Accession number(s).

This whole-genome shotgun project has been deposited in DDBJ/EMBL/GenBank under the accession and version numbers listed in Table 1.
